# Temperature and host plant influence alate formation in the hedgehog grain aphid, *Sipha maydis* (Hemiptera: Aphididae)

**DOI:** 10.1093/jee/toag110

**Published:** 2026-04-27

**Authors:** Rafael Hayashida, Rodrigo Marubayashi, Norman C Elliott, William Wyatt Hoback

**Affiliations:** Department of Entomology and Plant Pathology, Oklahoma State University, Stillwater, OK, USA; Department of Statistics, Londrina State University, Londrina, PR, Brazil; USDA-ARS, Plain Area, Oklahoma and Central Plains Research Center, Peanut and Small Grains Research Unit, Stillwater, OK, USA; Department of Entomology and Plant Pathology, Oklahoma State University, Stillwater, OK, USA

**Keywords:** wing polymorphism, *Sipha maydis*, aphid–host interaction, aphid polymorphism

## Abstract

The hedgehog grain aphid (HGA), *Sipha maydis* Passerini (Hemiptera: Aphididae), is a significant pest of cereal crops and is present in the United States. Like other aphids, it exhibits wing polymorphism, producing both winged and apterous morphs influenced by environmental factors such as temperature and host plant quality. This study evaluated the effects of temperature and host plant species on life history traits and wing induction under controlled laboratory conditions. Two weeks after nymphs being placed on host plants, a significant interaction between host plant and temperature was observed for number of aphids and winged morph proportion. The highest aphid populations occurred on wheat and barley at 30 °C (356 ± 70 and 344 ± 42, respectively), while sorghum supported consistently lower populations, peaking at 15 °C (37 ± 4). Wing induction was greatest on sorghum and barley at 25 °C (26.9 ± 7.5% and 10.1 ± 1.6%, respectively), while no differences were observed among temperatures on wheat (x̄ = 9.2 ± 2.0%), suggesting that the host may have induced a stress-related morphological response to promote dispersal, particularly on sorghum, the least suitable host. Supercooling points (SCPs) of winged and apterous adults reared at 25 °C were also measured. While SCPs did not differ significantly between morphs, they varied by host plant: aphids reared on wheat froze at −16.7 °C, compared to −18.7 °C on sorghum and −18.6 °C on barley, indicating host-mediated cold tolerance. These findings underscore the ecological plasticity of HGA and provide valuable insights for improving outbreak prediction and integrated pest management strategies across diverse environmental conditions.

## Introduction

The hedgehog grain aphid (HGA), *Sipha maydis* Passerini (Hemiptera: Aphididae), is a significant agricultural pest that causes economic losses globally, especially in south temperate regions (Wieczorek and Bugaj-Nawrocka 2014). Although its economic importance has been considered limited in some northern regions, its capacity to colonize subtropical areas and inflict damage on cereal crops is increasingly recognized (Hammon 2015, Mornhinweg et al. 2020, Lampert et al. 2023). Worldwide, HGA is considered a cereal pest with an extensive geographical range and has been documented feeding on 53 plant host species, including important cultivated crops such as wheat (*Triticum aestivum*), sorghum (*Sorghum bicolor*), and barley (*Hordeum vulgare*), as well as various wild grasses (Puterka et al. 2019, Taylor et al. 2023a).

HGA is believed to be native to Europe, the Middle East, Asia, and parts of Africa (Puterka et al. 2019). In South America, HGA was first detected in Argentina in 2002, and it quickly spread across the region, leading to substantial economic losses in wheat (Corrales et al. 2007). In the United States, HGA was initially detected in giant wildrye (*Leymus condensatus*) in California in 2007 (Sorensen and Center 2007), and since then, it has been monitored due to its potential to damage crops. Its presence has been recorded across various states in the United States, including Colorado, New Mexico, Utah, Wyoming, and Florida, indicating its adaptability to diverse climatic conditions (Halbert et al. 2013, Puterka et al. 2019, Taylor et al. 2023a).

Aphids exhibit remarkable phenotypic plasticity; on secondary host plants, they are able to produce both winged (females viviparae and gynoparae and males) and apterous viviparae morphs from the same genotype, a phenomenon known as wing polymorphism (Moran 1992, Dixon 1997, Brisson 2010). This plasticity aids survival and dispersal, allowing aphid populations to locate host plants and environmental conditions that allow survival and reproduction (Deem et al. 2024). The development of winged morphs is influenced by a complex interplay of environmental and genetic factors, as well as associations with microorganisms (Nunes and Hardie 1996, Brisson 2010, Zhang et al. 2015, Rozo‐Lopez and Parker 2023).

The development of winged morphs is strongly influenced by unfavorable environmental conditions, including tactile stimulation resulting from high population densities and declining host plant quality (Müller et al. 2001, Parker et al. 2021). These factors often interact in complex ways to induce wing formation. For instance, aphids feeding on more nutritious hosts tend to exhibit a higher fecundity rate, leading to faster population growth and, consequently, an earlier initiation of crowding-related cues for wing formation (Müller et al. 2001).

Temperature, as a key abiotic factor, significantly affects aphid physiology and the development of host plants, thereby shaping aphid demographics. Aphids generally develop faster at warmer temperatures to an upper limit at which they die. A correlation between average air temperature and HGA flight activity has been observed as evidenced by the presence of winged HGA in the fields (Lampert et al. 2023), but the temperature at which this occurs and the influence of host plant are unknown. Aphids can also survive temperatures below the threshold for development. However, at extremely cold temperatures, death occurs when the body fluids freeze, which is known as the supercooling point (SCP). The SCP is defined as the temperature at which an insect’s body fluids crystallize, releasing latent heat (Armstrong et al. 1998, Lee 2010).

Despite the current limited economic impact of HGA in the United States, continuous monitoring is imperative because of its broad host range and potential for significant economic damage (Halbert et al. 2013, Taylor et al. 2023a). Better understanding of the environmental conditions that promote the development of winged forms of HGA is critical for more effective monitoring and accurate modeling of its potential distribution and future outbreaks. Additionally, determining the SCP for each HGA morph reared on different crops can provide valuable insights for predicting HGA overwintering.

The objectives of this study were to evaluate the interaction between temperature (15, 20, 25, and 30 °C) and host plant species (wheat, barley, and sorghum) on the development of wings by HGA under controlled laboratory conditions. Additionally, we determine the SCP of apterous and winged HGA individuals reared on each host plant.

## Materials and Methods

### Insect and Plant Materials

The HGA colonies were initially collected from crested wheatgrass (*Agropyron cristatum*) in Taos, New Mexico, in 2019. Colonies are maintained on “Yuma” wheat in 4.4 liters pots fitted with Lexan cylinders (45 cm tall × 16 cm diameter; SABIC Polymershapes, Tulsa, Oklahoma, United States) and covered with organdy cloth tops to prevent aphid escape. The HGA colonies are housed in an APHIS-approved quarantine facility at the USDA-ARS station in Stillwater, Oklahoma, to prevent introduction, as HGA have not been documented in wild populations in Oklahoma (Puterka et al. 2019, Taylor et al. 2023a).

The quarantine facility is maintained at a consistent temperature of 21 ± 2 °C with a 14:10 h light:dark photoperiod, using seven TS 32 W Ecolux daylight fluorescent lamps (Fairfield, Connecticut, United States) as the light source. To maintain colony health and viability, aphids were transferred to fresh host plants weekly or as needed when plant quality deteriorated.

### Influence of Host Plant Species and Temperature on Life History and Formation of Wings

The experiment was conducted three times, each lasting 30 d: the first beginning on 13 April 2024; the second on 14 November 2025; and the third on 4 December 2025. For the first 2 experimental runs, four identical controlled-environment growth chambers (Model DS72SD, Powers Scientific Inc., Warminster, Pennsylvania), each capable of independent temperature and light control, were used. In the third run, a different set of chambers (Model E30B, Percival, Perry, Iowa, United States) was used. During all runs, chambers were programmed to maintain constant temperatures of 15, 20, 25, or 30 ± 0.5 °C, with a consistent 16:8 light:dark photoperiod to evaluate the effects of temperature and host plant species on aphid life-history traits and wing formation.

Within each chamber, 4.4 liter pots were prepared with a 3-layered growth medium, following methods by Taylor et al. (2023b). The medium consisted of a base layer of potting soil, followed by a layer of clay, and topped with sand. Ten seeds were sown in each pot. Following germination, seedlings were thinned to retain five uniform plants per pot. Three host plant genotypes, all known to be susceptible to HGA, were employed: wheat (*Triticum aestivum* cv. Jagger), barley (*Hordeum vulgare* line 812), and sorghum (*Sorghum bicolor* line TX7000) (Taylor et al. 2023b). The experiment followed a split-plot design, where temperature was assigned as the whole-plot factor (growth chambers) and host plant species was assigned as the split-plot factor (pots within each chamber). Temperature treatments were randomly assigned to growth chambers in each run, and host plant arrangements were randomized within each chamber. Each treatment combination (host plant × temperature) was replicated ten times, with one pot representing a single replicate. For the experimental setup, ten apterous adult aphids were introduced to 2-wk-old plants in each replicate. After 2 wk of infestation, the following parameters were recorded: the total number of aphids, the number of winged individuals, and the proportion of winged forms (%), which was subsequently calculated.

### Supercooling Point Determination

To determine the SCP of the apterous and winged forms of HGA, 3 colonies were maintained on barley, wheat, and sorghum as described above, with constant temperature of 25 ± 0.5 °C. Each colony was initiated with 10 individuals and maintained for 40 d to ensure a sufficient number of individuals for testing. Individual apterous and winged adult aphids were affixed to a 30-gauge copper-constantan thermocouple coated with a thin layer of petroleum jelly (Walmart Inc., Bentonville, Arkansas, United States). The thermocouple was connected to a CR12x micrologger (Campbell Scientific, Logan, Utah, USA) and placed inside a Pyrex test tube (2.5 × 15 cm). The test tube was held upright and partially submerged in a cooling bath composed of approximately 0.5 kg of dry ice and 0.75 liter of 70% ethyl alcohol, following Armstrong et al. (1998).

The SCP experiment was conducted on 15 October 2024. The cooling bath temperature was maintained within a range of −30 °C and −35 °C. The exposure temperature for the aphids was regulated by gradually lowering the thermocouple and aphid into the test tube. The temperature decreased at a rate of approximately 2.5 °C per minute, and the temperature readings were recorded every 0.2 s using the data logger. Each insect measurement took about 8–10 min. The SCP was identified as the lowest body temperature reached by the aphid before the release of latent heat due to crystallization, a phenomenon clearly observable in the temperature time series data. Apterous and winged adults reared on the three host plants (wheat, barley, and sorghum) were tested. Each individual aphid was treated as a single replicate, with twenty replicates performed for each host plant-morph combination.

### Statistical Analysis

Data from the first experiment, in which we evaluated the influence of host plant species and temperature on life history and wing formation, were analyzed using a Generalized Linear Mixed Model (GLMM) assuming a Poisson distribution for total counts and a binomial distribution for proportion data. The model included the fixed effects of temperature, plant species, and their interaction, and the random effects of block and block × temperature. The significance of effects was assessed using Type II deviance analysis (Wald test), and multiple comparisons were performed using Tukey’s test at a 5% significance level.

For the SCP experiment, the SCPs of each individual aphid were determined, and the average SCP for each host plant and morph was calculated. These average SCPs were then compared using a two-way ANOVA (host plant × morph). Before proceeding with the two-way ANOVA, we performed exploratory data analysis to assess the assumptions of normality of residuals (Shapiro and Wilk 1965) and homogeneity of variances (Burr and Foster 1972). The means were subsequently analyzed using a two-way ANOVA with a significance level of α = 0.05, and multiple comparisons were performed using Tukey’s test at a 5% significance level. Statistical analyses were performed within the R computing environment, utilizing the “AgroR” package (Shimizu et al. 2022) for the analyses. Graphs were designed using the “Ggplot2” package (Wickham 2016).

## Results

### Effect of  Temperature and Host Plant on HGA Wing Formation

**Fig. 1.**
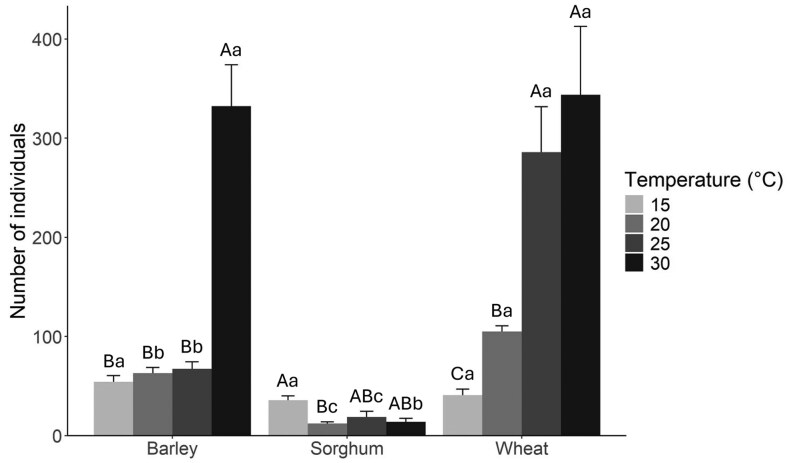
Effect of temperature and host plant on *Sipha maydis* (hedgehog grain aphid; HGA) population growth. Bars (means ± SE) with the same uppercase letter within each host plant, and lowercase letters within each temperature, do not differ significantly according to Tukey’s test (α = 0.05).

A significant interaction between host plant and temperature was observed for the total number of aphids [χ^2^(6) = 119.28, *P *< 0.001; Fig. 1]. In barley, the highest number of individuals was observed at 30 °C, with an average ± SE of 344.41 ± 41.57 individuals. No difference was observed among the other temperatures with means varying from 58.31 ± 5.94 to 69.89 ± 6.65 individuals. In wheat, the highest number of individuals was observed at 25 °C and 30 °C, with 296.30 ± 46.54 and 356.48 ± 70.32, respectively, and the lowest number was observed at 15 °C with 40.86 ± 5.95 individuals. In contrast, in sorghum, the highest number of individuals was observed at 15 °C with 36.89 ± 4.39 and the lowest number of individuals was observed at 20 °C, with 13.00 ± 1.72 (Fig. 1). Overall, wheat and barley showed the highest number of individuals across all tested temperatures (356.48 and 344.41, respectively), while sorghum had the lowest (13.00), indicating it may not be the best host plant [χ^2^(2) = 271.11, *P *< 0.001; Fig. 1].

**Fig. 2.**
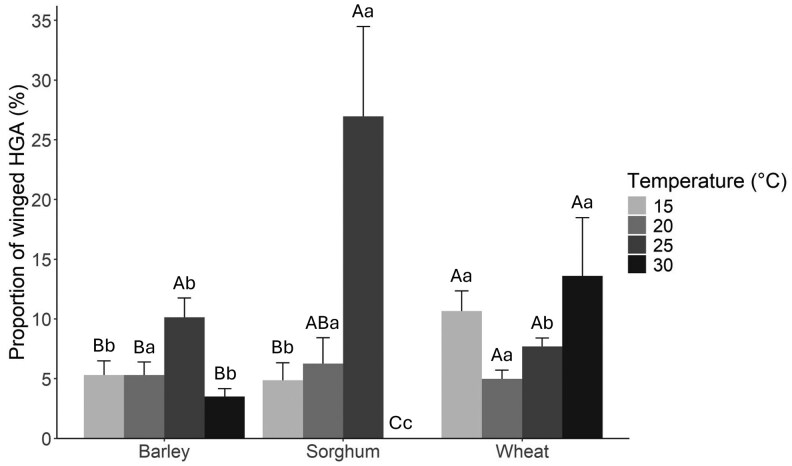
Effect of temperature and host plant on wing formation in *Sipha maydis* (hedgehog grain aphid; HGA). Bars (means ± SE) with the same uppercase letter within each host plant, and lowercase letters within each temperature, do not differ significantly according to Tukey’s test (α = 0.05).

The proportion of winged aphids was also influenced by the interaction of the host plant and temperature [χ^2^(6) = 18.68, *P* < 0.001; Fig. 2]. On barley, the highest proportion was observed at temperatures of 25 °C, with 10.18 ± 1.63%, and no differences were observed among the other temperatures, varying from 5.31 ± 1.09% to 3.51 ± 0.67%. On sorghum, the highest proportion was observed at 25 °C, with 26.95 ± 7.53% of individuals producing wings. No winged aphids were observed at 30 °C on sorghum (Fig. 2). The proportion of winged aphids on wheat did not differ across any of the temperatures tested (Fig. 2).

### Supercooling Point of HGA Reared on Different Hosts

**Fig. 3.**
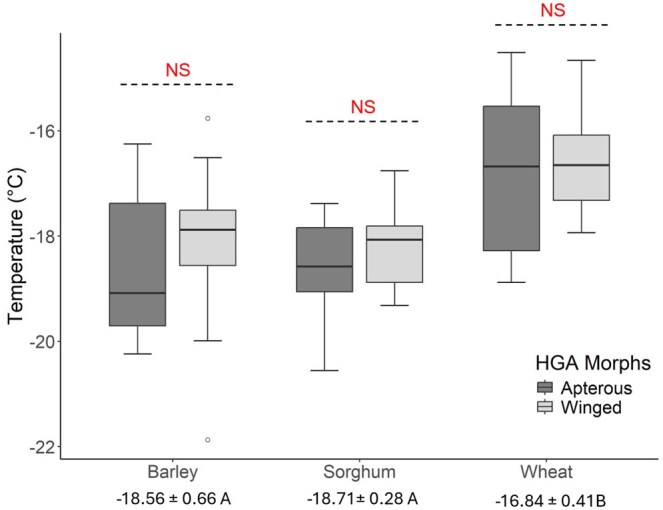
Supercooling point (± SE, °C) of apterous and winged morphs of the hedgehog grain aphid (HGA), *Sipha maydis*, reared on three different host plants under laboratory conditions (21 ± 2 °C, 14:10 L:D). Means ± SE represent the SCP of individuals reared on each host plant. Means followed by the same letters do not differ significantly according to Tukey’s test (α  =  0.05); NS, no significant differences between HGA morphs.

The two-way ANOVA test revealed no significant interaction between host plant and wing presence [*F*(2, 62) = 0.035, *P* = 0.96], and no significant main effect of wing presence [*F*(1, 62) = 1.18, *P* = 0.28]. However, there was a significant main effect of host plant [*F*(2, 62) = 14.73, *P* < 0.001; Fig. 3] on SCP. Individuals reared on wheat exhibited a higher SCP (–16.84 °C) compared to those reared on sorghum and barley (–18.56 °C and –18.71 °C, respectively).

## Discussion

The findings of this study provide important information for predicting HGA outbreaks and better understanding host plant use, which are essential for developing effective management tactics. Our results demonstrate that both temperature and host plant significantly influence HGA population growth and formation of winged individuals, while host plant can also affect the SCP.

The observed variations in total aphid numbers across different host plants and temperatures demonstrate the importance of environmental factors in shaping HGA population growth. For instance, the highest populations on wheat at 25 °C and 30 °C, and on barley at 30 °C, indicate the most favorable conditions for HGA development on each host at those temperatures. This pattern suggests that barley supports better aphid performance at higher temperatures, whereas wheat may provide more suitable conditions under moderate temperatures. Further research should evaluate whether barley maintains its suitability as a host under even higher temperature regimes. Conversely, the lower populations of HGA occurred on sorghum across all the tested temperatures, except at 15 °C, where no differences were detected among host plants, indicating that sorghum may be a less favorable host plant.

While Taylor et al. (2023b) observed that HGA population parameters are affected by temperature when reared on wheat, they did not find significant differences in the life table parameters when HGA was offered sorghum, wheat, or millet as host plants at 23 °C. This contrasts with our findings, in which we detected clear differences in the number of individuals and in the proportion of winged morphs among the host plants tested. This discrepancy may be explained by the fact that the HGA population tested by Taylor et al. (2023b) differed from the population evaluated in our study. Overall, the findings of our study align with previous research indicating that temperature and host plant directly influence HGA population demographics (Tazerouni and Talebi 2014, La Rossa et al. 2017, Lampert et al. 2023).

Our study also reveals that the proportion of winged forms is significantly influenced by the interaction between host plant and temperature, for some plants. The highest proportions of winged individuals were observed on barley and sorghum at 25 °C, whereas no differences were detected on wheat across all tested temperatures. This pattern suggests that HGA responds differently to temperatures on different hosts by producing dispersal morphs. Interestingly, when HGA was reared on sorghum at 25 °C, it exhibited one with the lowest total number of individuals but the highest proportion of winged forms. This observation supports that wing polymorphism in aphids is a key adaptive trait facilitating dispersal, especially under unfavorable conditions such as a poor host plant (Brisson 2010). While environmental cues, such as deteriorating host quality or suboptimal temperatures, typically trigger alate formation, enabling aphids to seek more suitable habitats (Müller et al. 2001, Parker et al. 2021), the absence of winged forms on sorghum at 30 °C is particularly noteworthy. This observation warrants further investigation, as it may indicate that the 2-wk infestation period was not sufficient for HGA to develop winged forms on sorghum (or even to build up a population at all), or that temperatures and a non-preferred host inhibited wing development.

In addition to poor abiotic conditions, tactile stimulation resulting from high population density is known to induce the production of winged forms in aphid (Deem et al. 2024). Under natural conditions, HGA populations can vary widely in density, ranging from small groups (5 to 20 individuals) to large aggregations (>50 individuals) (Taylor et al. 2023a). In our two-week experiment, the number of individuals per pot often exceeded 100 aphids, which may have contributed to the cues triggering wing formation. However, tactile stimulation alone does not fully explain this response, as we did not observe a direct relationship between the highest aphid densities and the highest proportions of winged forms in each temperature-host plant combination (Figs 1 and 2). In fact, aphids on wheat at 25 °C and 30 °C, and barley at 30 °C, showed the highest population densities but the lowest percentages of winged individuals (Figs 1 and 2), indicating that these cereals offer more suitable conditions than sorghum. Although Taylor et al. (2023b) reported a lack of resistance to HGA in sorghum (including TX7000, the same genotype tested here), our results suggest that sorghum still performs poorly as a host under the tested conditions.

While no differences were detected in the SCP between apterous and winged HGA, individuals reared on different hosts had different SCPs, with lower values observed in aphids from sorghum and barley (–18.71 and –18.56 °C, respectively). Previous work has reported similar SCP for HGA nymphs, apterous adults, and winged adults reared on wheat variety “Jagger,” ranging from –15.30 to –16.55 °C (Taylor et al. 2023b). The SCP is primarily determined by an organism’s inherent traits, such as body composition, which are closely associated with its physiological condition, including factors like feeding status, diapause, developmental stage, and metamorphosis (Renault et al. 2002). Host plants appear to influence the cold tolerance of HGA, potentially through nutritional differences that affect the insect’s body composition. It is important to note that aphid mortality by low temperatures is not determined by SCP alone and the exposure duration and environmental conditions also play critical roles (Armstrong and Peairs 1996, Puterka et al. 2019).

In conclusion, this study provides valuable data for developing more accurate predictive models of HGA outbreaks and distribution, ultimately contributing to more effective and sustainable management strategies. This is particularly important given the limited presence of natural enemies controlling HGA in the field (Corrales et al. 2007, Puterka et al. 2019) and the limited known genetic resistance in major crops (Corrales et al. 2007). The role of wings in HGA dispersal to avoid harmful temperatures, along with plant associations and availability of hosts on which to overwinter should be considered in forecast models where HGA could become a serious economic pest in the way that sorghum aphid, *Melanaphis sorghi* caused widespread economic losses between 2012 and the present (Bowling et al. 2016, Harris-Shultz et al. 2019).
